# Comparison of chronic widespread pain prevalence with different criteria in two cohorts of rheumatoid arthritis

**DOI:** 10.1007/s10067-021-05999-8

**Published:** 2021-11-23

**Authors:** M. Aronsson, S. Bergman, E. Lindqvist, M. L. E. Andersson

**Affiliations:** 1 Section of Rheumatology, Department of Clinical Sciences Lund, Lund University, Lund, Sweden; 2 Spenshult Research and Development Center, Halmstad, Sweden; 3 Department of Rheumatology, Capio Movement, Halmstad, Sweden; 4 School of Public Health and Community Medicine/Primary Health Care, Institute of Medicine, Sahlgrenska Academy, University of Gothenburg, Gothenburg, Sweden; 5 Department of Rheumatology, Skåne University Hospital, Lund, Sweden

**Keywords:** Arthritis, Chronic pain, Lifestyle, Pain, Physical activity, Rheumatoid

## Abstract

**Objective:**

This study aims to investigate chronic widespread pain with the 1990 (CWP1990) and 2019 (CWP2019) definitions 6 years after the onset of rheumatoid arthritis (RA), in one patient cohort with tight controls and one conventional cohort, and factors associated with reporting CWP1990 and CWP2019, respectively.

**Methods:**

A cohort of 80 RA patients with monthly visits to the physician the first 6 months was compared to a cohort of 101 patients from the same clinic with conventional follow-up. Both cohorts had early RA (< 13 months). The prevalence of CWP1990 and the more stringent CWP2019 were in a 6-year follow-up investigated with a questionnaire, including a pain mannequin and a fear-avoidance beliefs questionnaire.

**Results:**

In the tight control cohort, 10% reported CWP2019 after 6 years compared to 23% in the conventional cohort (*p* = 0.026). There was no difference when using the CWP1990 definition (27% vs 31%, *p* = 0.546). When adjusted for important baseline data, the odds ratio for having CWP2019 was 2.57 (95% CI 1.02–6.50), in the conventional group compared to the tight control group (*p* = 0.046). A high level of fear-avoidance behaviour towards physical activity was associated with CWP2019, OR 10.66 (95% CI 1.01–112.14), but not with CWP1990 in the tight control cohort.

**Conclusion:**

A more stringent definition of CWP identifies patients with a more serious pain condition, which potentially could be prevented by an initial tight control management. Besides tight control, caregivers should pay attention to fear-avoidance behaviour and tailor treatment.

**Key Points:**

• *CWP2019 is a more stringent definition of chronic widespread pain and identifies patients with a more serious pain condition.*

• *Patients with a serious pain condition could be helped by frequent follow-ups.*

• *This study suggests that a special attention of fear-avoidance behaviour towards physical activity in patients with RA is needed.*

## Introduction

Chronic musculoskeletal pain, lasting 3 months or more, is common in the adult general population [1, 2]. The concept of chronic widespread pain (CWP), initially part of the 1990 criteria for fibromyalgia, is defined as pain lasting 3 months or more located axially, in the right and left side of the body and above and below the waist, and will in this article be referred to as CWP1990 [3]. Around 10% of the general population around the world suffers from CWP [1, 2, 4, 5]. The CWP1990 definition has been criticized since it could be fulfilled with as few as 3 painful sites (for example, pain in the back, right leg and left arm), making it possible to misclassify patients with regional pain as CWP [3, 6]. Considering this, a new more stringent definition of CWP has been developed, WP2019, which requires pain in at least 4 of 5 body regions (left upper, left lower, right upper, right lower, axial) and at least 7 painful sites out of 15[7].

For fibromyalgia (FM), defined with the 1990 criteria (CWP1990 and 11 of 18 tender pressure points), the estimated prevalence in the general population is around 2% [2]. More recent definitions of FM have abandoned the tender point examination and instead rate the widespreadness of pain and somatic symptoms connected to the disease such as fatigue and cognitive symptoms [8–10].

It is well-known that patients with rheumatoid arthritis (RA) often suffer from concomitant CWP and FM, with prevalence numbers around 30% for CWP1990 and 20% for FM [11–14]. Studies have shown that persistent pain in RA patients remains despite good control of inflammation and modern pharmacological treatment options and that RA patients have an increased risk of developing FM [11, 15–17].

To our knowledge no studies have investigated CWP and FM prevalence in RA patients that have received tight control regimen early in the disease. There are some data supporting an association between a high level of fear-avoidance behaviour regarding physical activity and higher pain levels in RA, but the possible connection to CWP in this group of patients has not been studied [18]. Furthermore, RA patients with moderate to high pain levels are less likely to fulfil the generally recommended dose of weekly physical activity (≥ 150 min moderately intense physical activity/week) [19]. Some studies have proposed an association between alcohol and CWP in the general population, with a possible protective effect of moderate alcohol intake, but this remains unclear and we have little knowledge of alcohol consumption and its potential connection to CWP in RA patients [20, 21].

The primary aim of this study was to investigate the prevalence of CWP1990, CWP2019 and FM 6 years after the onset of rheumatoid arthritis and compare two patient cohorts, one with tight control and one with conventional follow-up early in the disease. The secondary aim was to explore factors associated with reporting CWP1990 and CWP2019, respectively.

## Materials and methods

### Patients

For this single-centre 6-year follow-up study, we recruited patients from two cohorts: the tight control cohort and the conventional BARFOT cohort [22]. The inclusion criteria were RA diagnosis with a symptom duration less than 13 months. The exclusion criteria were change of diagnosis. No patients were excluded. The tight control cohort consisted of early RA patients included between 2011 and 2016, which were monitored with monthly follow-ups by a physician during the first 6 months and thereafter at 9, 12 and 24 months. Clinical and patient-reported data were collected at each visit. The conventionally managed patients from the BARFOT cohort, used in this study, were early RA patients included between 2001 and 2006. They were followed every 3 months the first 6 months and thereafter at 12 and 24 months. Clinical and patient-reported data were collected at each visit.

### Questionnaire

The patients in the conventional cohort were sent a questionnaire in 2010, in median 6 (IQR 4.5–7.0) years after disease onset. The response rate was 83%. The questionnaire contained questions about pain and fatigue on a visual analogue scale (VAS) and comorbidity including patient-reported diagnosis of FM. Additionally, the Swedish version of Stanford Health Assessment Questionnaire (HAQ) [23–25] to measure patient function and the EuroQol 5 dimensions (EQ-5D) to measure health associated quality of life [26, 27] was included. Pain distribution was reported on a pain mannequin [1].

The patients in the tight control cohort were sent a questionnaire in 2019–2020, in median 6 (IQR 4.0–7.0) years after disease onset. The response rate was 74%. The questionnaire contained the same questions as the questionnaire used in 2010 mentioned above. Additionally, it had a Fear-Avoidance Beliefs Questionnaire (FABQ) [18, 28], an Alcohol Use Disorders Identification Test-Consumption (AUDIT-C) [29] and questions regarding time spent each week on moderate and vigorous physical activity [30].

### Chronic widespread pain (CWP)

In this study, we use two definitions of CWP, CWP2019 based on the new WP2019 definition [7] and CWP1990 based on the well-established definition from ACR1990 criteria for FM [3]. The pain mannequin used in the questionnaires was developed to assess CWP according to the 1990 criteria and included the following body sites, chest, neck, thoracic spine, lumbar and sacral spine, and, additionally, left and right, shoulder and upper arm, lower arm, hand, upper leg, knee, lower leg and foot. One point per site gives a score range of 0–18. In order to adopt our mannequin as close as possible to the WP2019 definition of CWP, we excluded the chest region and the knees from the calculations. This enabled us to have 5 main pain regions with 3 sites in each, giving a score range of 0–15 in the same way as the original WP2019 [7]. Since the calculation was not exactly the same, we here use the term CWP2019 instead of WP2019. CWP2019 was calculated as pain ≥ 3 months in at least 4 of the 5 main body regions proposed in WP2019 (the upper and lower quadrants of the body and the spine) and at least 7 painful sites out of 15 possible. The CWP1990 was in accordance with the ACR1990 criteria calculated from the pain mannequin as ≥ 3 months of pain in the left and right side of the body, above and below the waist as well as axially [3].

### Fear-Avoidance Beliefs Questionnaire (FABQ)

The Fear-Avoidance Beliefs Questionnaire, originally developed to evaluate back pain but thereafter used for many patient groups including RA, consists of two subscales [18, 19, 28, 31]. The first subscale is related to physical activity (FABQPA) and based on 4 items about physical activity causing pain and injury. Ratings on each item are made on a 7-point scale 0–6, and the total score range is 0–24. The second subscale is related to work and is not used in this study; however, the participants filled in the complete FABQ. The median FABQPA in the tight control cohort was 8; therefore, a value of 8 or above was considered a high level of FABQPA in this study. A previous study of RA patients in Sweden and FABQPA demonstrated a comparable median FABQPA of 7 and used > 7 as a cut-off for high FABQPA[19].

### Alcohol Use Disorders Identification Test-Consumption (AUDIT-C)

The AUDIT-C is a short version of AUDIT with 3 questions connected to alcohol consumption. The total score range is 0–12, and a total score ≥ 4 in women and ≥ 5 in men is considered a risk consumption of alcohol [29].

### Physical activity

The questions used for physical activity were taken from the 2016 Swedish national health survey and included questions on the number of minutes spent each week on moderate and vigorous activity [30].

### Ethics and consent

All patients signed a written consent to participate in the study. Ethical approval was obtained for the tight control cohort (LU 2012/604, LU2018/824) and for the conventional cohort (LU 398–01) from the regional ethical review board at Lund University, Lund, Sweden. The study followed the guidelines from the Helsinki Declaration, and the management of the database was according to ISO 9001.

### Statistical methods

Statistical analyses were carried out using SPSS Statistics 26 software, IBM, USA. The significance level in the analyses was set to < 0.05, and non-parametric methods were used since many of the variables were not normally distributed. The normality of the data was tested with Shapiro–Wilk normality test. Comparisons were made between patients with and without CWP2019 at baseline using Mann–Whitney *U* test and Pearson’s chi-square test. For continuous variables, the median value has been given with the first and third quartiles, as dispersion measures. When *n* < 5 in any of the groups, Fisher’s exact test was used instead of chi-square. Regarding the questionnaire data from the 6-year follow-up, the tight control cohort and the conventional cohort were compared using Mann–Whitney *U* test and chi-square test. Patients fulfilling the CWP1990 criteria were compared to patients fulfilling CWP2019 criteria. The groups were compared by clinical baseline data and questionnaire data with Mann–Whitney *U* test and Pearson’s chi-square test.

To investigate factors associated with fulfilment of CWP1990 criteria and CWP2019 criteria at 6-year follow-up, a multiple regression controlled for study cohort, gender, age, disease duration and VAS pain at inclusion was also performed. To investigate the association of FABQPA, AUDIT-C and physical activity on CWP2019 in the tight control cohort, univariate logistic regressions were performed with these variables as well as gender, age, disease duration and VAS pain at inclusion. Variables with *p* < 0.3 were then included in multiple logistic regressions for having CWP2019 at follow-up [32]. Thereafter, the same multiple logistic regression was repeated for having CWP1990 at 6-year follow-up.

## Results

### Demographic and clinical characteristics at baseline

Tables 1 and 2 show baseline data for the two cohorts: the tight control cohort with 80 patients and the conventional cohort with 101 patients. Data in each table is presented for the whole cohort and split into patients fulfilling CWP2019 or not at 6-year follow-up, respectively. In the tight control cohort, the patients that later fulfilled CWP2019 at follow-up had significantly less swollen joints at baseline. The difference was not significant in the conventional cohort. In the conventional cohort, the patients that later fulfilled CWP2019 had lower CRP at baseline; the same pattern could not be seen in the tight control cohort. There was no significant difference in VAS pain at baseline between patients that later fulfilled CWP2019 or not in either of the cohorts.

**Table 1:** Baseline characteristics for all patients in the tight control cohort, as well as divided into groups of patients with and without CWP2019 at the 6-year follow-up. Presented values are median (q1–q3) or numbers (%). *p* values are comparisons between the group fulfilling CWP2019 and those not fulfilling CWP2019

	Missing	All tight control *n* = *80*	Tight control with CWP2019 *n* = *8*	Tight control no CWP2019 *n* = *71*	*p* value
Age, years	0	64 (54–71)	66 (49–72)	63 (54–70)	0.852
Gender, female	0	51 (64)	6 (75)	44 (62)	0.469
Disease duration months	0	5 (3–7)	3 (1–6)	5 (3–7)	0.058
Ever smoker	0	52 (65)	7 (88)	44 (62)	0.152
RF positive	0	49 (61)	6 (75)	43 (61)	0.425
ACPA positive	9	48 (68)	5 (83)	43 (67)	0.657*
DAS 28 (0–9.4)	3	4.64 (3.86–5.48)	4.69 (3.96–5.40)	4.67 (3.72–5.68)	0.993
Swollen joint count (0–28)	0	5 (3–8)	3 (2–4)	6 (3–9)	0.024
Tender joint count (0–28)	0	5 (2–10)	3 (2–9)	5 (2–10)	0.344
CRP (mg/L)	2	15.5 (7.3–33.0)	15.50 (9.25–78.08)	15.20 (7.0–33.0)	0.616
ESR (mm/h)	1	23 (11–38)	33 (15–70)	23 (11–37)	0.242
HAQ (0–3)	5	1.0 (0.63–1.38)	1.0 (0.78–1.47)	1.0 (0.60–1.38)	0.463
VAS pain (0–100)	4	56 (33–80)	68 (48–81)	56 (32–80)	0.299
PGA no	**	0	0	0	
PGA low		9 (12)	1 (14)	8 (12)	
PGA moderate		41 (55)	4 (57)	37 (55)	0.966
PGA high		24 (32)	2 (29)	22 (33)	

*CWP2019*, chronic widespread pain according to 2019 definition; *IQR*. interquartile range; *RF*, rheumatoid factor; *ACPA*, anti-citrullinated peptide antibody; *DAS28*, 28-joint Disease Activity Score; *CRP*, C-reactive protein; *ESR*, erythrocyte sedimentation rate; *HAQ*, Health Assessment Questionnaire; *VAS*, visual analogue scale. *PGA*, physicians’ global assessment of disease activity. *Fisher’s exact test. **5 missing PGAs

**Table 2:** Baseline characteristics for all patients in the conventional BARFOT as well as divided into groups of patients with and without CWP2019 at the 6-year follow-up. Presented values are median (q1–q3) or numbers (%). *p* values are comparisons between the group fulfilling CWP2019 and those not fulfilling CWP2019

	Missing	All Conventional BARFOT *n* = *101*	Conventional BARFOT with CWP2019 *n* = *23*	Conventional BARFOT no CWP2019 *n* = *78*	*p* value
Age, years	0	58 (45–68)	54 (45–70)	58 (45–67)	0.833
Gender, female	0	66 (65)	18 (78)	48 (62)	0.139
Duration, months	0	5 (4–8)	7 (4–9)	5 (4–8)	0.504
Ever smoker	4	48 (50)	13 (59)	35 (47)	0.305
RF positive	2	60 (61)	13 (59)	47 (61)	0.869
ACPA positive	27	51 (69)	12 (63)	39 (71)	0.529
DAS 28 (0–9.4)	7	5.18 (4.44–6.24)	4.79 (3.95–5.99)	5.26 (4.52–6.27)	0.159
Swollen joint count (0–28)	1	8 (4–11)	6 (3–9)	8 (4–12)	0.063
Tender joint count (0–28)	1	8 (4–14)	7 (3–12)	8 (4–14)	0.367
CRP (mg/L)	0	15.0 (7.0–34.0)	9.0 (7.0–20.0)	19.0 (7.0–38.5)	0.04
ESR (mm/h)	0	25 (11–40)	20 (7–29)	26 (14–41)	0.09
HAQ (0–3)	4	1.0 (0.63–1.50)	1.07 (0.75–1.53)	0.88 (0.63–1.50)	0.238
VAS pain (0–100)	5	49 (30–68)	44 (29–69)	49 (32–68)	0.947
PGA no	*	0	0	0	
PGA low		17 (17)	8 (35)	9 (12)	
PGA moderate		59 (60)	13 (56)	46 (61)	0.017
PGA high		23 (23)	2 (9)	21 (28)	

*CWP2019*, chronic widespread pain according to 2019 definition; *Age*. age at inclusion; *Duration*, duration at inclusion; *IQR*, interquartile range; *RF*, rheumatoid factor; *ACPA*, anti-citrullinated peptide antibody; *DAS28*, 28-joint Disease Activity Score; *CRP*, C-reactive protein; *ESR*, erythrocyte sedimentation rate; *HAQ*, Health Assessment Questionnaire; *VAS*, visual analogue scale. *PGA*, physicians’ global assessment of disease activity. *2 missing PGAs

### Prevalence of CWP and FM

The prevalence of CWP after 6 years was lower in the tight control cohort where 10% had CWP2019 compared to 23% in the conventional cohort (*p* = 0.026). For CWP1990, the prevalence was 27% in the tight control cohort and 31% in the conventional cohort (Table 3). The self-reported prevalence of FM was 0% in the tight control cohort and 5% in the conventional cohort at 6-year follow-up (*p* = 0.058) (Table 3).

**Table 3:** Comparison of self-reported survey data for the two cohorts, 6 years from inclusion. Presented values are median (q1–q3) or numbers (%). *p* values are comparisons between the tight control group and the conventional group (BARFOT)

	Missing Tight control/conventional	Tight control *n* = *80*	Conventional (BARFOT) *n* = *101*	*p* value
CWP1990	1/0	21 (27)	31 (31)	0.546
CWP2019	1/0	8 (10)	23 (23)	0.026
Fibromyalgia	10/1	0 (0)	5 (5)	0.058
VAS pain (0–100)	3/2	20 (0–40)	30 (20–50)	0.012
VAS fatigue (0–100)	2/0	30 (10–50)	40 (20–65)	0.113
HAQ (0–3)	0/0	0.0625 (0.0–0.625)	0.375 (0.0–0.813)	0.018
EQ-5D (0–1)	1/6	0.796 (0.725–0.850)	0.727 (0.725–0.796)	0.222
*CWP1990*				
CWP		21 (27)	31 (31)	
CRP	1/0	42 (53)	50 (49)	0.827
NCP		16 (20)	20 (20)	
*CWP2019*				
CWP		8 (10)	23 (23)	
CRP	1/0	54 (68)	58 (57)	0.081
NCP		17 (22)	20 (20)	

*CWP*, chronic widespread pain; *CWP1990*, dichotomized CWP according to 1990 definition; *CWP2019*, dichotomized CWP according to 2019 definition; *VAS*, visual analogue scale 0–100 mm; *HAQ*, Health Assessment Questionnaire; *EQ-5D*, EuroQol 5 dimensions; *CRP*, chronic regional pain; *NCP*, no chronic pain

### Pain, function, quality of life and fatigue

The tight control cohort reported less pain at 6 years with a median VAS pain of 20 (q1–q3, 0–40) compared to 30 (q1–q3, 20–50) in the conventional cohort (*p* = 0.012). The HAQ was also significantly lower in the tight control cohort with a median of 0.0625 compared to 0.375 in the conventional cohort (*p* = 0.018) (Table 3). There were no significant differences in EQ-5D and VAS fatigue between cohorts (Table 3).

### Pharmacological treatment

At inclusion, there were no differences in pharmacological treatment between the groups (*p* = 0.852). No patients were treated with bDMARD, and 69% vs. 71% in the tight control group and conventional group were treated with methotrexate, respectively. The patients did not report their pharmacological treatment in the questionnaire at 6-year follow-up. However, the clinical data from the 2-year follow-up shows that 84% of patients in the tight control cohort and 86% in the conventional cohort had methotrexate as a part of their pharmacologic treatment. Similarly, 8% of the tight control cohort and 9% of the conventional cohort were on bDMARD at the 2-year follow-up. More combination therapy with csDMARDs was used in the tight control cohort with 21% of patients on combination therapy vs 8% in the conventional cohort (*p* = 0.010). The tight control cohort also received more glucocorticoids at 2 years, 39% vs 18% in the conventional cohort (*p* = 0.002).

### Comparisons between patients with CWP1990 and CWP2019

More patients in the two cohorts fulfilled the liberal CWP1990 (*n* = 52) than the more stringent CWP2019 (*n* = 31) (Table 4). There was a substantial overlap of patients that met both definitions criteria. When analysing baseline data and follow-up data for the CWP1990 and the CWP2019 patients, no significant differences were found (Table 4). The patients fulfilling the CWP2019 criteria had at 6-year follow-up VAS pain median 50 (q1–q3, 40–70) vs. those not fulfilling the criteria that had 40 (30–60) (*p* = 0.073) (Table 4).

**Table 4:** Baseline data and self-reported survey data at 6 years, for patients from both cohorts fulfilling CWP1990 and CWP2019, respectively. Presented values are median (q1–q3) or numbers (%). *p* values are comparisons between the group fulfilling CWP1990 and those fulfilling CWP2019

	Missing CWP1990/CWP2019	CWP1990 *n* = *52*	CWP2019 *n* = *31*	*p* value
Baseline data				
Gender, female	0/0	40 (77)	24 (77)	0.958
Age, years	0/0	58 (48–70)	55 (47–70)	0.817
Duration, months	0/0	6 (3–8)	5 (3–8)	0.955
Ever smoker	1/1	33 (65)	20 (67)	0.858
RF positive	1/1	31 (61)	19 (63)	0.820
ACPA positive	10/6	28 (67)	17 (68)	0.911
Tender joint count (0–28)	0/0	7 (2–11)	6 (2–11)	0.861
Swollen joint count (0–28)	0/0	6 (3–8)	5 (3–8)	0.876
HAQ (0–3)	3/1	1.0 (0.75–1.5)	1.0 (0.75–1.5)	0.772
VAS pain (0–100)	4/2	56 (40–73)	52 (33–73)	0.501
DAS28 (0–9.4)	4/2	4.86 (3.92–5.74)	4.79 (3.95–5.74)	0.987
CRP (mg/L)	1/0	10.0 (7.0–29.0)	10.0 (7.0–21.0)	0.969
ESR (mm/h)	0/0	21 (8–40)	20 (8–40)	0.884
Follow-up 6 years				
VAS pain (0–100)	1/0	40 (30–60)	50 (40–70)	0.073
VAS fatigue (0–100)	0/0	60 (40–80)	60 (40–80)	0.70
Fibromyalgia	2/1	3 (6)	1 (3)	0.596
HAQ (0–3)	0/0	0.688 (0.375–1.125)	0.750 (0.375–1.250)	0.744
EQ-5D (0–1)	2/1	0.725 (0.620–0.796)	0.708 (0.603–0.727)	0.584
AUDIT-C alcohol risk consumption	8/4	11 (25)	6 (22)	0.790

*CWP1990*, chronic widespread pain according to 1990 definition; *CWP2019*, chronic widespread pain according to 2019 definition; *Age*, age at inclusion; *Duration*, disease duration at inclusion; *IQR*, interquartile range; *RF*, rheumatoid factor; *ACPA*, anti-citrullinated peptide antibody; *HAQ*, Health Assessment Questionnaire; *VAS*, visual analogue scale; *DAS28*, 28-joint Disease Activity Score; *CRP*, C-reactive protein; *ESR*, erythrocyte sedimentation rate; *EQ-5D*, EuroQol 5 dimensions; *AUDIT-C alcohol risk consumption*, AUDIT-C ≥ 4 for women and ≥ 5 for men

### Factors associated with CWP1990 and CWP2019 at follow-up

To investigate factors associated with having CWP1990 and CWP2019 at 6-year follow-up, multiple logistic regressions were performed with patients from both cohorts. The odds ratio for patients in the conventional cohort of having CWP2019 was 2.57 (95% CI 1.02–6.50), compared with the patients in the tight control cohort (Table 5). Gender, age, disease duration and VAS pain at inclusion had no significant association to CWP2019 (Table 5). No significant associations were found for having CWP1990 (Table 5).

**Table 5:** Factors associated with having CWP1990 and CWP2019 at 6-year follow-up in the two cohorts analysed with multiple logistic regression

	CWP1990			CWP2019		
	No	OR (95% CI)	*p* value	No	OR (95% CI)	*p* value
*Cohort*						
Tight control cohort	75	1		75	1	
Conventional cohort	96	1.24 (0.60–2.56)	0.56	96	2.57 (1.02–6.50)	0.046
Male gender	171	0.48 (0.22–1.03)	0.059	171	0.56 (0.22–1.44)	0.23
Age, years	171	1.0 (0.98–1.03)	0.89	171	1.0 (0.97–1.03)	0.98
Duration, months	171	0.99 (0.88–1.11)	0.87	171	0.94 (0.81–1.08)	0.36
Inclusion VAS pain, 0–100 mm	171	1.01(1.0–1.02)	0.16	171	1.01 (0.99–1.02)	0.56

*CWP1990*, chronic widespread pain according to 1990 definition; *CWP2019*, chronic widespread pain according to 2019 definition; *Age*, age at inclusion; *Duration*, disease duration at inclusion; *VAS*, visual analogue scale

### Additional factors associated with CWP2019 in the tight control cohort

A high score (≥ the median 8) on the FABQPA, indicating a fear-avoidance behaviour concerning physical activity at follow-up, was associated with also having CWP2019 but not with having CWP1990. In the multivariate model, the odds ratio for patients with high FABQPA to have CWP2019 was 10.66 (95% CI 1.01–112.14, *p* = 0.049) when adjusted for alcohol use, duration and VAS pain at inclusion (Table 6). When performing the same multivariate model for having CWP1990, there were no significant associations for FABQA, alcohol use, duration or VAS pain at inclusion. Risk consumption of alcohol and physical activity showed no significant association with CWP1990 nor CWP2019.

**Table 6:** Factors associated with having CWP2019 at 6-year follow-up in the tight control cohort (univariate logistic regression and multiple logistic regression)

	Model 1, univariate logistic regression for having CWP2019			Model 2, multivariate logistic regression for having CWP2019		
	No	OR (95% CI)	*p* value	No	OR (95% CI)	*p* value
Gender	79	0.54 (0.10–2.89)	0.47			
Age, years	79	1.0 (0.94–1.06)	0.99			
Duration, months	79	0.75 (0.54–1.03)	0.074	59	0.82 (0.60–1.14)	0.24
Inclusion VAS pain 0–100 mm	75	1.02 (0.99–1.05)	0.26	59	1.02 (0.97–1.06)	0.46
High FABQPA, 0–24	75	8.13 (0.95–69.76)	0.056	59	10.66 (1.01–112.14)	0.049
High AUDIT-C, 0–12	63	3.06 (0.62–15.18)	0.17	59	4.67 (0.70–31.14)	0.11
Moderate physical activity ≥ 150 min/week	52	0.66 (0.13–3.28)	0.61			

*CWP2019*, chronic widespread pain according to 2019 definition; *Age*, age at inclusion; *Duration*, disease duration at inclusion; *VAS*, visual analogue scale; *High FABQPA*, ≥ 8 on the Fear-Avoidance Beliefs Questionnaire on Physical Activity; *High AUDIT-C*, AUDIT-C ≥ 4 for women and ≥ 5 for men indicating risk consumption

## Discussion

In this 6-year follow-up study, patients in a tight control cohort with monthly clinical visits the first 6 months had less CWP2019 6 years later than patients in a conventionally managed cohort. The odds ratio for having CWP2019 after 6 years if participating in the conventional cohort was more than twice as high compared to being in the tight control cohort, when adjusted for gender, age, disease duration and VAS pain at inclusion. The same pattern could not be seen in the odds ratio for CWP1990. The more stringent CWP2019 criteria include patients with more widespread pain since CWP in the older 1990 definition can be fulfilled with as little as 3 painful regions. Pain can affect physical function, but there were no differences in HAQ between those reporting CWP2019 or not in this study. However, worse physical function could be caused by pain with and without inflammation [33]. There are also studies reporting that FM interferes with disease activity and thereby also with treatment outcomes both cross-sectional and longitudinal [34, 35].

Interestingly, no patients in the tight control cohort reported a diagnosis of FM at 6-year follow-up, although previous studies have shown a prevalence of FM around 20% in RA patients [12, 13]. However, the reported FM prevalence in the conventional cohort of 5% was also lower than expected, probably because of the self-reported approach. Maybe, the 23% of patients, with a true widespread pain, fulfilling the CWP2019 in the conventional cohort more corresponds to the around 20% FM that could be expected. In this case, a tight control management initially could have a protective effect. These findings raise the question is there a window of opportunity for development of CWP in RA?

Many studies have shown that early intensive treatment is important for the achievement of remission, but can the same apply for development of chronic pain [36, 37]? In a longitudinal study from 2019, Larrosa Pardo et al. show that RA patients have an increased risk of developing CWP and fibromyalgia over time compared to patients without RA and hypothesizes about the role of central sensitization in patients with an underlying painful condition [16]. It is possible that an early remission in a tight control program leads to less pain and therefore avoids triggering central sensitization. Previous studies have shown that compliance to the pharmacological treatment can be higher in tight control management [36]. However, it is known that also psychological factors such as fear of pain, the patient’s sense of safety and confidence in the treatment as well as their perception of pain can influence chronic pain development [38, 39]. Possibly, these factors are an important explanation why the tight control cohort in our study experienced less CWP2019 after 6 years, since it is probably easier to convey a sense of security and reassurance to the patients seen regularly at a monthly basis. The fact that a high level of fear-avoidance behaviour towards physical activity was associated with CWP in our study additionally stresses the importance of the rehabilitation team in the care of early RA patients. The study also shows that a more stringent classification of CWP can be justified when evaluating the presence of widespread pain. The two classification criteria capture partly different groups of patients, where the patients fulfilling the CWP2019 can be considered to have a more serious widespread pain problem.

The limitations of the present study are of course the lack of randomization to tight control or conventional follow-up, as well as the fact that the two cohorts were included during different time frames. This could potentially affect their pharmacological treatment, but at least at the 2-year follow-up, both cohorts had similar proportions of patients on methotrexate and bDMARD. In this study, we only had baseline pain in the form of VAS pain. It would have been an advantage to have a baseline record of CWP to better describe development of CWP longitudinally in the two cohorts.

The strengths of the present study are the fact that we have two cohorts of early RA patients, both included during the biological era of RA treatment and both followed at the same clinic, but with different follow-up schemes the first 6 months. Both cohorts received a questionnaire with the same pain mannequin to investigate the prevalence of CWP and fibromyalgia 6 years after disease onset. To our knowledge, CWP development in tight control management of early RA has not been studied previously.

There is a need for future research in the field of CWP, especially longitudinally studies to investigate further what can be done to prevent CWP and fibromyalgia in RA patients, especially since more recent studies in the biological era demonstrates that chronic pain is still a problem for many RA patients [14, 40]. These chronic pain problems have the capacity to affect both the patients’ quality of life and their ability to work.

To conclude, we here demonstrate that tight control management of patients with early RA have a positive potential to reduce the prevalence of the more stringent CWP2019 and that RA patients with a fear-avoidance behaviour towards physical activity are at increased risk of having CWP2019. Early efforts by the rehab team to promote physical activity in patients with a fear-avoidance behaviour could be a valuable addition to a tight control management of early RA patients.

## Data Availability

The datasets used and/or analysed during the current study are available from the corresponding author on reasonable request.

## Code availability

Exemplar code for a sample analysis can be provided upon request to the corresponding author.
